# Low-Dose Nivolumab with or without Ipilimumab as Adjuvant Therapy Following the Resection of Melanoma Metastases: A Sequential Dual Cohort Phase II Clinical Trial

**DOI:** 10.3390/cancers14030682

**Published:** 2022-01-28

**Authors:** Julia Katharina Schwarze, Soizic Garaud, Yanina J. L. Jansen, Gil Awada, Valérie Vandersleyen, Jens Tijtgat, Alexandre de Wind, Paulus Kristanto, Teofila Seremet, Karen Willard-Gallo, Bart Neyns

**Affiliations:** 1 Department of Medical Oncology, Vrije Universiteit Brussel (VUB)/Universitair Ziekenhuis Brussel (UZ Brussel), Laarbeeklaan 101, 1090 Brussels, Belgium; gil.awada@uzbrussel.be (G.A.); valerie.vandersleyen@uzbrussel.be (V.V.); jens.tijtgat@uzbrussel.be (J.T.); teofila.caplanusi@chuv.ch (T.S.); bart.neyns@uzbrussel.be (B.N.); 2 Molecular Immunology Unit, Institut Jules Bordet, Université Libre de Bruxelles, 1000 Brussels, Belgium; soizic.garaud@bordet.be; 3 Department of Thoracic Surgery, Vrije Universiteit Brussel (VUB)/Universitair Ziekenhuis Brussel (UZ Brussel), Laarbeeklaan 101, 1090 Brussels, Belgium; yaninajansen@gmail.com (Y.J.L.J.); karen.willard-gallo@bordet.be (K.W.-G.); 4 Department of Pathology, Institut Jules Bordet, Université Libre de Bruxelles, 1000 Brussels, Belgium; roland.dewind@bordet.be; 5 Data Center, Institut Jules Bordet, Université Libre de Bruxelles, 1000 Brussels, Belgium; paulus.kristanto@bordet.be

**Keywords:** adjuvant, melanoma, immunotherapy, low dose, nivolumab, ipilimumab

## Abstract

**Simple Summary:**

Optimal dosing and duration of adjuvant treatment with PD-1 immune checkpoint inhibitors in melanoma patients have not been established. The investigated low-dose regimen of nivolumab with or without ipilimumab (in a sequential dual-cohort phase II trial), resulted in a 12-months relapse-free survival (RFS) rate and tolerability that was comparable to what has been served with standard dosing of nivolumab or pembrolizumab when patients were matched for stage. The incidence of immune-related adverse events was similar to what has been reported from registration trials in this indication. Immunohistochemical quantification of intra- and peritumoral immune cells, but not PD-1/PD-L1 staining, correlated significantly with RFS. Therefore, low-dose regimes of PD-1 blocking monoclonal antibodies deserve further study as cost-effective alternatives for currently approved standard dosing regimens, with baseline immunohistochemical tumor profiling to be further explored as a promising biomarker.

**Abstract:**

Background: Optimal dosing and duration of adjuvant treatment with PD-1 and CTLA-4 immune checkpoint inhibitors have not been established. Prior to their regulatory approval we investigated a low-dose regimen of nivolumab with or without ipilimumab in a sequential dual-cohort phase II clinical trial. Methods: Following the complete resection of melanoma metastases, patients were treated with a single fixed dose of ipilimumab (50 mg) plus 4 bi-weekly fixed doses of nivolumab (10 mg) (cohort-1), or nivolumab for 1 year (10 mg fixed dose, Q2w x9, followed by Q8w x4) (cohort-2). Twelve-months relapse-free survival (RFS) served as the primary endpoint. Results: After a median follow-up of 235 weeks for cohort-1 (34 patients), and 190 weeks for cohort-2 (21 patients), the 12-months RFS-rate was, respectively, 55.9% (95% CI, 39–72), and 85.7% (95% CI, 70–100). Treatment-related adverse events occurred in 27 (79%), and 18 (86%) patients, with 3 (9%), and 1 (5%) grade 3 adverse events in cohort-1 and -2, respectively. Immunohistochemical quantification of intra- and peritumoral CD3^+^ T cells and CD20^+^ B cells, but not PD-1/PD-L1 staining, correlated significantly with RFS. Conclusions: One year of adjuvant low-dose nivolumab could be an effective and economically advantageous alternative for standard dosing, at the condition of further confirmation in a larger patient cohort. A shorter low-dose nivolumab plus ipilimumab regimen seems inferior and less tolerable.

## 1. Introduction

Melanoma patients with resectable metastases are at high risk of disease recurrence after definitive surgery [1]. In 2015, based on the results of the European Organisation for Research and Treatment of Cancer (EORTC) 18,071 phase III trial, the US Food and Drug Administration (FDA) approved ipilimumab, a cytotoxic T-lymphocyte-associated antigen 4 (CTLA-4)-blocking monoclonal antibody (mAb), for the adjuvant treatment of stage III melanoma patients with regional lymph node metastasis of >1 mm after complete resection [2]. In this trial, ipilimumab at a dose of 10 mg per kilogram every 3 weeks for four administrations, then every 3 months for up to 3 years or until disease recurrence or an unacceptable toxicity occurred was compared to placebo [2]. At 5 years of follow-up, ipilimumab resulted in a significantly improved relapse-free survival (RFS), distant metastasis-free survival (DMFS), and overall survival (OS). However, treatment was associated with 42% of grade 3 or 4 immune-related adverse events (irAE), and 1.1% of grade 5 irAE. In 2019, a significant improvement of RFS was obtained with the programmed cell death protein 1 (PD-1) blocking mAb nivolumab (CheckMate-238 phase III trial) or pembrolizumab (EORTC 1325 phase III trial) when compared to ipilimumab or placebo, respectively. Moreover, irAE were significantly lower with either anti-PD-1 mAb [3]. As a result, anti-PD-1 immune checkpoint inhibitors (ICI) became the preferred adjuvant immuno-oncology treatment for melanoma patients [3,4]. Additional evidence for the superiority of pembrolizumab over high-dose interferon or ipilimumab was generated by the results of the intergroup S1404 phase III randomized trial [5]. The U.S. Intergroup E1609A phase III randomized study of adjuvant ipilimumab (3 or 10 mg/kg) versus high-dose interferon alfa-2b for resected high-risk melanoma established superiority of the ipilimumab 3 mg/kg regime [6]. For both anti-PD-1 mAb, dosing mirrored their standard dosing regimens in the unresectable setting with nivolumab being administered at a dose of 3 mg per kilogram every 2 weeks, and pembrolizumab at 200 mg every 3 weeks. Recently, results of the CheckMate-915 phase III trial indicated that one year of therapy with nivolumab (240 mg every 2 weeks) plus ipilimumab (1 mg/kg every 6 weeks) failed to improve RFS of patients with resected stage IIIB-D and stage IV melanoma as compared to nivolumab monotherapy (480 mg every 4 weeks) [7]. Similar to data in advanced melanoma patients treated with the combination therapy, the incidence of irAE was also significantly increased with the combination in the adjuvant setting (33% grade ≥3 irAE, and 0.4% grade 5 AE for the combination regimen) [7].

Prior to their approval in the adjuvant setting, nivolumab, pembrolizumab, and ipilimumab had significantly improved progression-free survival (PFS) and OS in patients with unresectable melanoma and were approved as a monotherapy, or as an upfront combination therapy (nivolumab plus ipilimumab) [8,9,10,11,12,13,14,15,16]. In patients responding to anti-PD-1 monotherapy or nivolumab/ipilimumab combination therapy, treatment duration was arbitrarily defined as “until progression” (nivolumab monotherapy or combination) or “a total of 2 years of therapy” (pembrolizumab) [11,17]. Observations from phase I clinical trials and real-world observational data on the outcome of patients who electively discontinued treatment with anti-PD-1 ICI clearly indicate that durable PFS can be obtained after stopping therapy [18,19,20]. These data suggest that a minimum exposure of 6-months may be recommended [18,21]. In the presence of treatment-limiting toxicity with the combination regimen, patients benefiting from treatment had equal PFS irrespective of whether they had to interrupt treatment or not within the first 12 weeks of drug exposure [22]. As for ipilimumab, duration of therapy in patients with unresectable melanoma has been limited to four 3-weekly doses. Activity of retreatment at the time of progression in patients who benefited from initial treatment has been demonstrated [23]. For the combination of nivolumab and ipilimumab in patients with advanced melanoma it is questionable whether four doses of ipilimumab are necessary [24]. A phase II multicenter study addressing this issue has shown that the first two doses of the combination of nivolumab and ipilimumab appear to drive the response efficacy and occurrence of side effects, suggesting a shorter treatment interval [24].

Interestingly, in a phase I clinical trial in patients with advanced melanoma, overall response rates (ORR) and irAE were seen at a dose range of 0.1–10 mg/kg when administered bi-weekly in a phase I trial in patients with advanced melanoma [20]. An “intra-patient” dose escalation to 1.0 mg/kg in five and six patients initially treated with 0.1 mg/kg and 0.3 mg/kg, respectively, did not result in clinical responses in any of these patients. Additionally, durable occupancy of the PD-1 receptor on circulating T lymphocytes was observed with varying doses of nivolumab [25]. In contrast, for ipilimumab monotherapy a clear dose-response correlation was established, while the dose-level of ipilimumab largely determines the incidence of irAE when combined with nivolumab but does not seem to determine activity [13,26,27,28]. Notably, all clinical trials investigating ICI in the adjuvant setting that led to regulatory approval demanded that patients underwent a completion lymph node dissection (CLND). Following the results of the prospective, randomized phase III Multicenter Selective Lymphadenectomy Trial (MSLT) II trial and the German Dermatologic Cooperative Oncology Group (DeCOG) trial, demonstrating that in patients with microscopic disease a CLND does not lead to improved survival, and this practice was abandoned [29,30]. A potential impact of this change in surgical care on the outcome of adjuvant medical treatment options has not been investigated.

In this prospective, sequential dual-cohort phase II clinical trial, that was conducted prior to the regulatory approval of nivolumab and pembrolizumab in the adjuvant setting of melanoma. We investigated a regimen of low-dose nivolumab (fixed dose of 10 mg) with or without one administration of low-dose ipilimumab (single fixed dose of 50 mg) as adjuvant therapy in patients with high-risk stage III or IV melanoma following the resection of all macro-metastases. We also investigated the prognostic value of tumor-infiltrating lymphocytes and PD-1/PD-L1 expression.

## 2. Materials and Methods

### 2.1. Patient Eligibility and Regulatory Approval

Patients aged 18 years or older with histologically confirmed melanoma with metastases to regional lymph nodes or distant metastases that had been surgically resected were eligible for this trial. A completion lymph node dissection (CLND) was not required. Disease stage was determined according to American Joint Committee on Cancer (AJCC), 8th edition. At the time of enrolment, patients needed to have no evidence of disease according to an assessment with whole body imaging with ^18^F-fluorodeoxyglucose-positron emission tomography/computed tomography (^18^F-FDG-PET/CT), within 4 weeks preceding recruitment. All patients needed to have an Eastern Cooperative Oncology Group (ECOG) performance status score of 0 or 1. Other key recruitment criteria included adequate organ function, serum lactate dehydrogenase (LDH) and C-reactive protein (CRP) value lower than the upper limit of normal (ULN), and an absolute lymphocyte count (ALC) higher than 1500/mm^3^. Main exclusion criteria included previous exposure to ICI; ocular or uveal melanoma; leptomeningeal metastases, a history of autoimmune disease, systemic use of glucocorticoids, and a malignancy other than melanoma without complete remission for at least 3 years. This trial was approved by the medical ethics committee by Universitair Ziekenhuis Brussel and is registered under ClinicalTrials.gov: NCT02941744. The trial was conducted in accordance with Good Clinical Practice guidelines as defined by the International Conference on Harmonization. All patients needed to provide written informed consent.

### 2.2. Study Design and Treatment Schedule

This is a single center, sequential dual-cohort, non-randomized phase II clinical trial. Patients were treated in two cohorts; in cohort-1 patients received ipilimumab 50 mg (fixed dose, 1 administration) plus nivolumab 10 mg (fixed dose) at the first administration followed by a maximum of 3 additional administrations of nivolumab 10 mg (bi-weekly). In cohort-2 patients received nivolumab 10 mg (fixed dose) bi-weekly for 9 administrations followed by 4 four-weekly administrations. Study drug administration was terminated at an earlier timepoint in case of unacceptable toxicity, melanoma recurrence, or patient’s withdrawal of consent. Recruitment to cohort-2 was closed prematurely following registration of nivolumab in the adjuvant setting by European Medicines Agency (EMA).

### 2.3. Trial end Points

The primary endpoint was the 12 months relapse-free survival rate (12 months-RFS rate). Secondary endpoints were safety, distant metastasis-free survival (DMFS) and overall survival (OS).

### 2.4. Assessments

Assessments for detecting melanoma recurrence were performed by clinical examination and ^18^F-FDG-PET/CT whole-body imaging at baseline, and every 16 weeks thereafter. Patients who did not have a CLND were followed up by alternating ^18^F-FDG-PET/CT and ultrasound of the draining lymph node region every 8 weeks.

Safety was assessed on a continuous basis at every visit; AE were classified for type, severity (grade) and frequency according to National Cancer Institute (NCI) Common Terminology Criteria for Adverse Events (CTCAE) version 4.0. The safety analysis includes all patients who received at least one dose of the study drug.

### 2.5. Analysis of Circulating Tumor-DNA

As an exploratory objective, baseline presence of *BRAF^V600^* and *NRAS^Q61/G12/G13^* mutant circulating tumor DNA (ctDNA) was assessed by quantitative polymerase chain reaction (qPCR) using the Idylla™ Platform with software version 26.0 (Biocartis, Belgium). The used technique has been elaborately described in a previous publication by our research group [31].

### 2.6. IHC Staining and Pathologic Assessment

Formalin-fixed paraffin-embedded (FFPE) tissue sections (4 μm) were immunohistochemically (IHC) stained for CD3/CD20 and PD-L1/PD-1 dual staining on a Ventana Benchmark XT automated staining instrument (Ventana Medical Systems, Oro Valley, AZ, USA). A detailed protocol for the dual IHC stains has been previously described [32]. For CD3/CD20 dual staining, the slides were incubated with the ready-to-use polyclonal rabbit anti-CD3 primary antibody (IR50361-2, Agilent, Santa Clara, CA, USA) and the ready-to-use mouse monoclonal anti-CD20 primary antibody (IR60461-2, Agilent). For PD-L1/PD-1 dual staining, the slides were incubated with the monoclonal rabbit anti-PD-L1 primary antibody (13684S, Cell Signaling Technology^®^, Danvers, MA, USA) and the monoclonal mouse anti-PD-1 primary antibody (ab52587, Abcam, Cambridge, UK). Scoring TIL infiltration, lymphocyte subpopulation markers, and their organization in aggregates and tertiary lymphoid structures (TLS) on IHC-stained tissues was performed by one well-trained pathologist who was blinded to the clinical and experimental data. Lymphocytes in direct contact with tumor cells were identified as intratumoral TIL and those at the junction between tumor and immune stroma along a 0.5 mm delimited zone as peritumoral TIL. For assessment of PD-L1 expression, the pathologist was instructed to score as the % tumor cells, and peritumoral cells (macrophages exhibited the strongest level of PD-L1 expression).

### 2.7. Objectives and Statistical Analysis

Descriptive statistics were used to characterize the patient population, treatment disposition, and safety. The primary endpoint was the 12-months RFS-rate. Secondary endpoints were safety, DMFS and OS. We considered our investigational treatment of insufficient interest if the 12-months RFS-rate was 60% or lower. The RFS-rate at 12-months of approximately 60% for the placebo control arm of the EORTC 18,071 phase III trial investigating ipilimumab at a dose of 10 mg/kg in patients who had resected regional lymph node–positive (stage III) melanoma with a high risk of recurrence served as a historical control. We considered the investigational regimen to be of sufficient interest if a 12-months RFS-rate of >80% is observed in our study population. Using a Fleming one-stage design with a probability of Type I Error (alfa) of 0.05 and a Power (1-beta) of 0.8, a sample size of 33 patients was required. Accordingly, at least 27 patients should be free from recurrence of their melanoma at 12 months following the initiation of study treatment in order to consider this experimental regimen to be of sufficient interest for further clinical study. The recruitment to cohort-2 was closed prematurely following registration of nivolumab in the adjuvant setting by EMA. Kaplan–Meier analyses were used to estimate the RFS and the OS curves (in weeks), also RFS and OS of each derived categorical variable. A non-parametric log-rank test was used to compare survival estimates. The *p*-value of each log-rank test is reported on the graph accordingly. The cut-off of lymphocyte subpopulations was determined by the median. Univariate Cox regressions have been performed for each variable to obtain the estimated hazard ratio (HR) with 95% CI between the two categories. Analysis was performed using SPSS Statistics version 27.0 (IBM, Chicago, IL, USA) and SAS version 9.4 (SAS Institute, Cary, NC, USA).

## 3. Results

### 3.1. Baseline Characteristics

**Figure A1:**
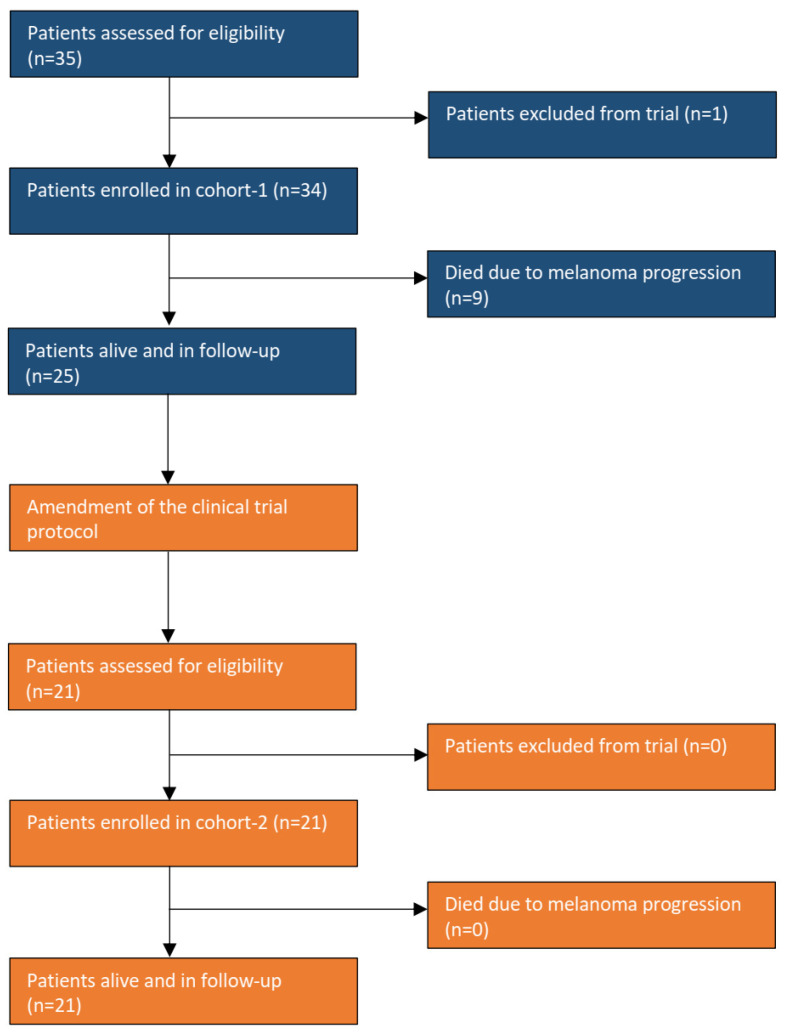
CONSORT-diagram. Patients enrolled to cohort-1 (prior to the trial amendment) are depicted in blue, patients enrolled in cohort-2 (after the trial amendment) are depicted in orange.

Between 20 April 2016 and 7 August 2017, 35 patients were screened, and 34 patients were enrolled in cohort-1; between 2 October 2017 and 2 August 2018, 21 patients were screened and enrolled in cohort-2 (Figure A1). Recruitment to cohort-2 was prematurely closed following EMA registration of nivolumab at a dose of 3 mg/kg in the adjuvant setting and subsequent reimbursement in Belgium in September 2018.

The median age was 54 (range 29–77), and 53 (range 36–78) years in cohort-1 and cohort-2, respectively. Fifteen patients (44%) and 10 patients (49%) were male in cohort-1 and -2, respectively.

Most patients had AJCC stage III disease and had undergone a complete resection of locoregional lymph node metastasis. Seventeen patients (50%) in cohort-1, and 5 patients (24%) in cohort-2 had been diagnosed with macroscopic nodal metastases; 2 patients (6%) in cohort-1 and 14 patients (67%) in cohort-2 had microscopic nodal metastases. Twelve patients (35%) and 2 patients (9%) in cohort-1 and cohort-2, respectively, underwent resection of in-transit or satellite metastases.

Three patients (9%), and one patient (4%) in cohort-1 and cohort-2, respectively, had undergone a resection of a solitary lung metastasis or distant subcutaneous metastasis (AJCC stage IV disease). A waiver was given to one patient in cohort-1 following the resection of a thick (pT4b), ulcerated primary melanoma without locoregional or distant metastasis (AJCC stage IIB).

Twenty patients (59%) in cohort-1 and 10 (48%) in cohort-2 had undergone a CLND before being enrolled in the trial. Fourteen (41%) and 11 patients (52%) in cohort-1 and -2 had undergone either a sentinel lymph node procedure only (*n* = 11 (32%) and *n* = 10 (48%)) or had undergone a resection of a distant metastasis (*n* = 3 (9%) in cohort-1 and *n* = 1 (4%) in cohort-2).

The melanoma of 15 patients (44%) in cohort-1, and 5 patients (24%) in cohort-2 were documented as *BRAF^V600^* mutant. Seventeen (50%) and 14 patients (67%) in cohort-1 and cohort-2, respectively, did not have a mutation in the *BRAF* gene. Ulceration of the primary tumor was present in 11 (32%) and 5 (24%) patients in cohort-1 and cohort-2, respectively.

An overview of these patient baseline characteristics is provided in Table 1.

### 3.2. Treatment Disposition

At the time of analysis, all enrolled patients were no longer receiving study treatment.

All patients in cohort-1 received a single dose of ipilimumab and 31 patients (91%) received all four scheduled nivolumab administrations. Treatment was prematurely stopped in three of patients (9%) due to toxicity (one patient received three doses, and two patients received two doses of nivolumab). In cohort-2, eight patients (38%) received all planned nivolumab administrations. The median number of treatment administrations was 12 (range 2–13). Four patients (19%) in cohort-2 temporarily interrupted study treatment due to toxicity, and four patients (19%) permanently stopped treatment due to toxicity. One patient stopped treatment due to melanoma recurrence; in two patients (10%) study treatment was stopped following patient’s withdrawal of consent (after two and nine study drug administrations), two patients preferred to switch to standard dosing of nivolumab following regulatory approval by EMA after, respectively, four and eight study drug administrations.

### 3.3. Safety

Adverse events of any grade that were considered to be related to the study treatment were observed in 27 (79%), and 18 (86%) patients in cohort-1 and cohort-2, respectively. Mainly low-grade adverse events were observed. The incidence of grade 3 adverse events was, respectively, 9% and 5% in cohort-1 and -2. There were no grade 4 or 5 adverse events.

The most frequent adverse event in both cohorts was fatigue (18 patients (53%) and 10 patients (48%) in cohort-1 and -2, respectively). The second most frequent were dermatological and endocrinological irAE; pruritus was observed in seven patients (21%; cohort-1) and eight patients (38%; cohort-2), rash in 8 patients (24%; cohort-1), and nine patients (43%; cohort-2). Six patients (18%) in cohort-1 developed hypothyroidism, preceded by hyperthyroidism in three patients. In cohort-2 two patients (10%) developed hypothyroidism, preceded by hyperthyroidism in one patient. Two patients (10%) in cohort-2 developed a grade 2 adrenal insufficiency caused by hypophysitis. Grade 3 irAE occurred in three patients (8%) and one patient (4%) in cohort-1 and cohort-2, respectively. Two patients (6%) in cohort-1 developed a grade 3 immune-related pneumonitis necessitating corticosteroid treatment. An additional patient developed a grade 3 polyarthritis. The only grade 3 irAE in cohort-2 was a cutaneous rash. All adverse events are listed in Table 2.

Table 2 Treatment-related adverse events according to National Cancer Institute Common Terminology Criteria for Adverse Events (CTCAE) version 4.0 (cohort-1; *n* = 34; cohort-2; *n* = 21). The safety analysis included all patients that received at least one dose of study treatment (*n* = 55). All adverse events observed between first dose administration until 30 days after the last dose administration are included.

### 3.4. Efficacy

#### 3.4.1. Recurrence-Free Survival

**Figure 1:**
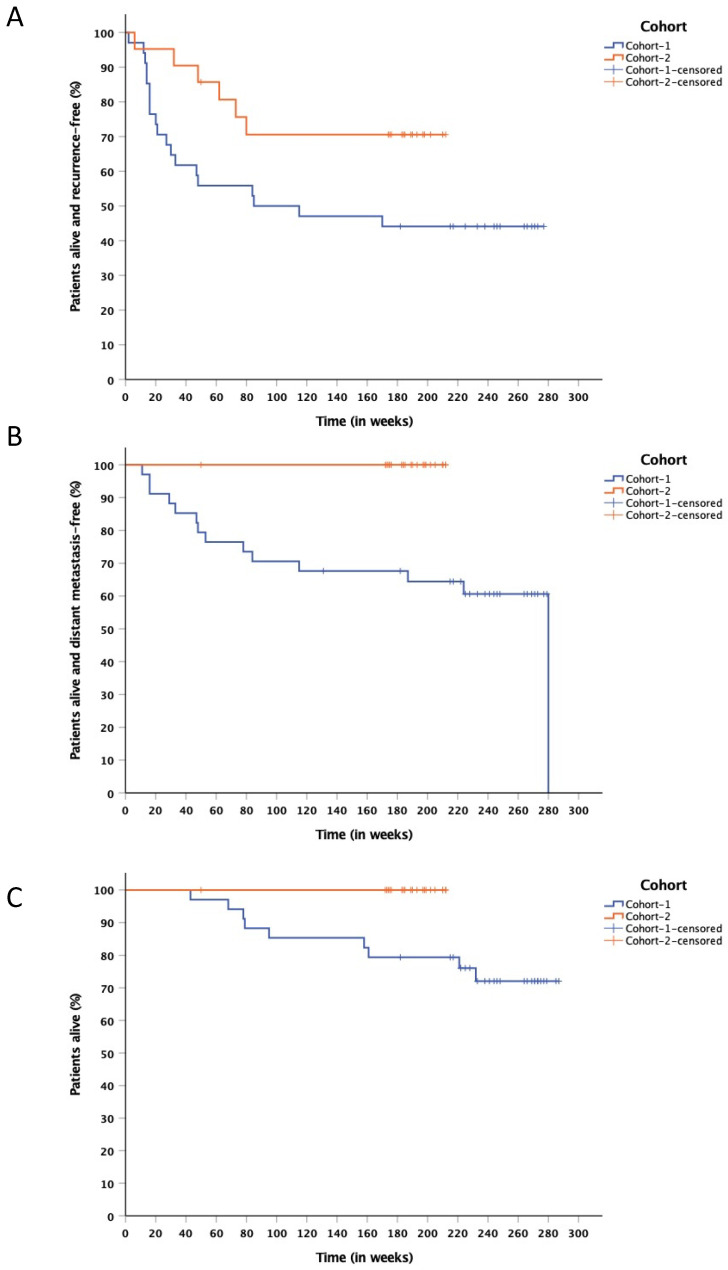
(**A**) Kaplan–Meier estimate of RFS in cohort-1 and cohort-2. At time of analysis, after a median follow-up of 235 (43–287) weeks for cohort-1 and 190 (50–210) weeks for cohort-2, estimated 12-months RFS-rate was, respectively, 55.9% (95% CI, 39–72) and 85.7% (95% CI, 70–100). Median RFS for cohort-1 was 85 weeks (95%, CI 0–254), not-reached in cohort-2. (**B**) Kaplan–Meier estimate of DMFS in cohort-1 and cohort-2. At time of analysis, 12-months DMFS-rate was 76.5% (95% CI, 62–90) in cohort-1. No distant metastases occurred in cohort-2. Median DMFS has not been reached at time of analysis in either cohort. (**C**) Kaplan–Meier estimate of OS in cohort-1 and cohort-2. After a median follow-up of 208 (43–256) weeks for cohort-1 and 157 (78–182) weeks for cohort-2, estimated 12-months OS-rate was, respectively, 97.1% (95% CI, 91–102) and 100%. Median OS has not been reached at time of analysis in either cohort.

After a median follow up of 235 (43–287) weeks for cohort-1, and 190 (50–210) weeks for cohort-2, 19 patients (56%) and 6 patients (29%) in cohort-1 and cohort-2, respectively, were diagnosed with recurrence of their melanoma. The estimated 12-months RFS-rate was, respectively, 55.9% (95% CI, 39–72) and 85.7% (95% CI, 70–100) (Figure 1A). Median RFS for cohort-1 was 85 weeks (95% CI, 0–254). Median RFS has not been reached for cohort-2. Subsequent anticancer therapy was administered in 19 patients (56%) in cohort-1 and in 6 patients (29%) in cohort-2. Two patients of cohort-1 developed clinical responses during treatment with an anti-PD-1 mAb for stage IV disease; another patient of cohort-1 achieved a complete remission after treatment with nivolumab and ipilimumab at standard dosing. In cohort-2, one patient who, after a second surgical resection of lymph node metastases, remained in complete remission after one year of treatment with nivolumab at standard dosing. Two patients of cohort-2 achieved a complete remission on combination treatment with ipilimumab and nivolumab.

#### 3.4.2. Distant Metastasis-Free Survival

The median DMFS was not reached in either cohort. Shorter DMFS was observed in cohort-1 with 14 patients (41%) having developed distant metastases; no patients in cohort-2 had developed distant metastases at time of analysis. The 12-months DMFS-rate was 76.5% (95% CI, 62–90) in cohort-1 (Figure 1B).

#### 3.4.3. Overall Survival

**Figure 2:**
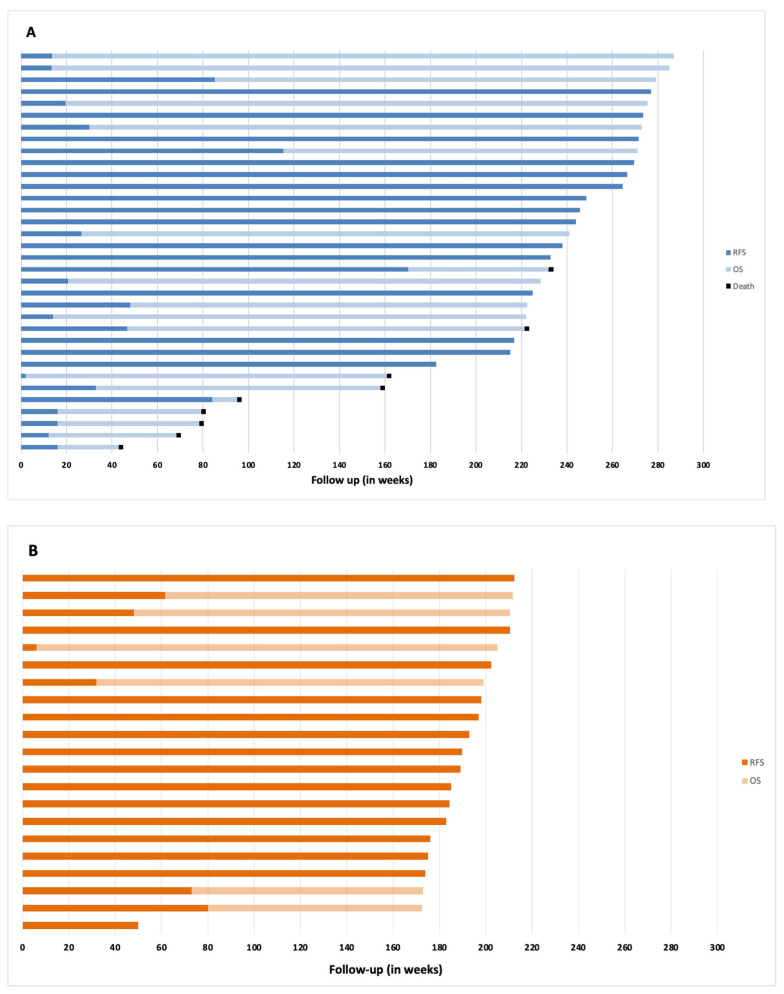
Swimmer plots representing duration of relapse-free survival (RFS) and overall survival (OS) of individual patients in cohort-1 (**A**) and cohort-2 (**B**). Dark blue bars and light blue bars depict RFS and OS, respectively. A black square at the end of a bar depicts death.

At time of analysis, 25 patients (73.5%) in cohort-1 are alive, and 9 patients have died due to melanoma; all 21 patients treated in cohort-2 are alive. After a median follow-up of 208 (43–256) weeks for cohort-1 and 157 (78–182) weeks for cohort-2, estimated 12-months OS-rate was, respectively, 97.1% (95% CI, 91–102) and 100% (Figure 1C). Median OS has not been reached in either cohort. The duration of RFS and OS per individual patient is represented in Figure 2.

### 3.5. The Prognostic Value of Tumor-Infiltrating Lymphocytes

**Figure 3:**
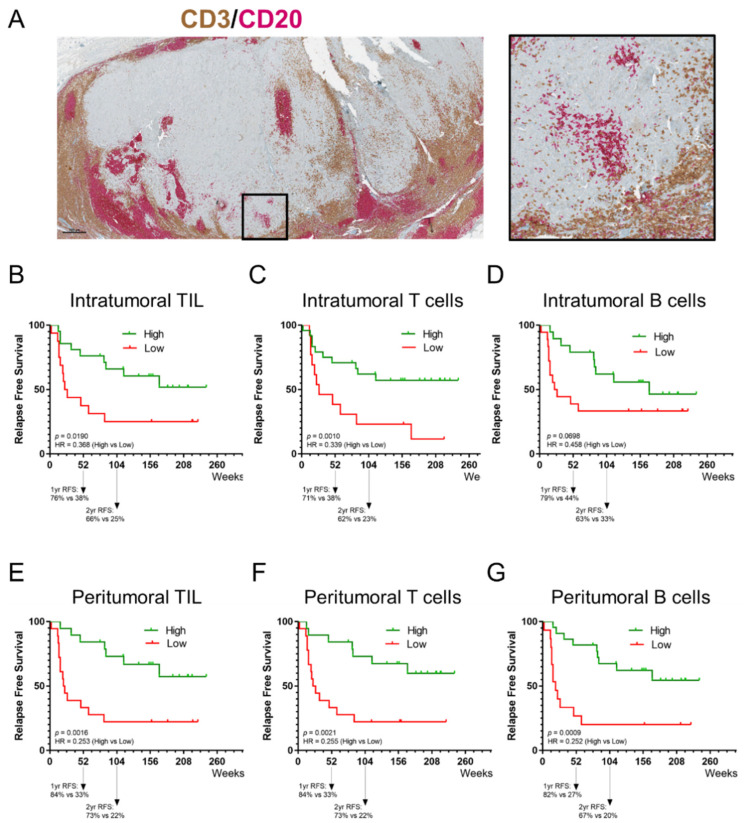
Prognostic value of tumor-infiltrating lymphocytes. (**A**) Representative image of a tissue section of a melanoma metastasis stained for CD3 and CD20; shown here are an overview of the section and a region of interest. IHC slides were scanned at ×40 magnification and the images are displayed at ×2.2 (Scale bar = 500 µm) and ×10 magnification. (**B**–**D**) Kaplan–Meier estimates of RFS for intratumoral TIL (both CD3^+^ T cells and CD20^+^ B cells), CD3^+^ T cells and CD20^+^ B cells. (**E**–**G**) Kaplan–Meier estimates of RFS for peritumoral TIL (both CD3^+^ T cells and CD20^+^ B cells), CD3^+^ T cells and CD20^+^ B cells. A higher intratumoral and peritumoral TIL, CD3^+^ T-cell, and CD20^+^ B-cell presence, except for intratumoral CD20^+^ B cells, were significantly associated with a longer RFS.

**Figure A2:**
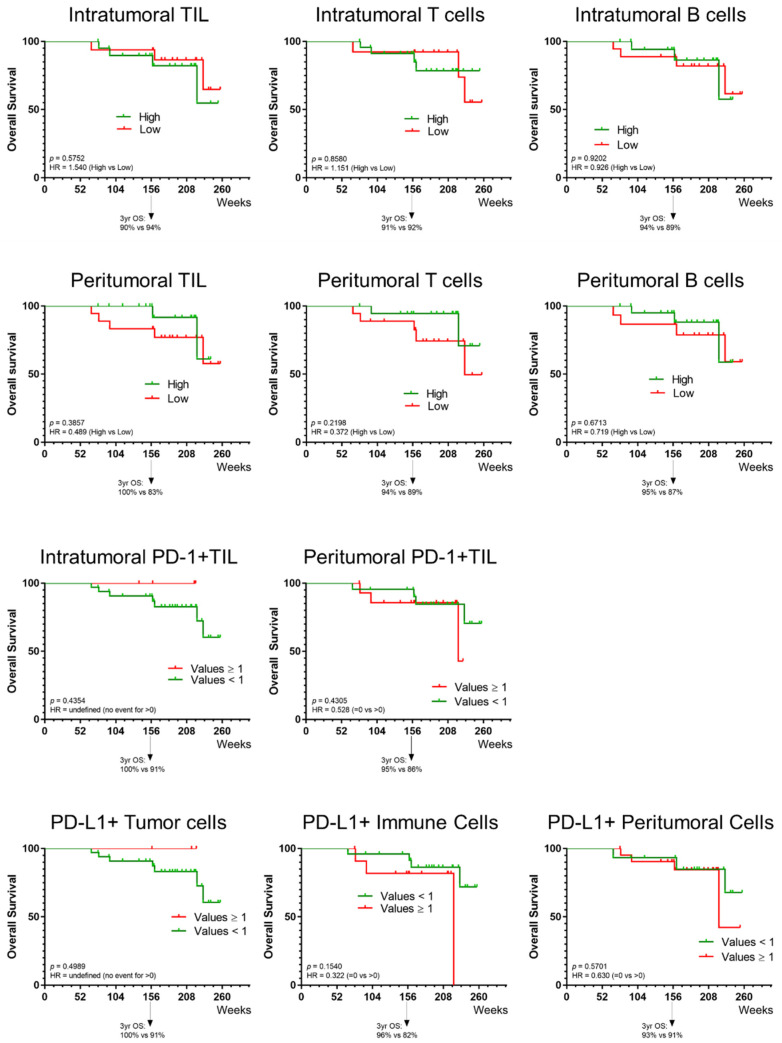
Tumor-infiltrating lymphocytes and prevalence of PD-1 and PD-L1 expression on cells in melanoma metastases and the potential correlation with survival outcomes. Kaplan–Meier estimates of OS for intratumoral TIL (both CD3^+^ T cells and CD20^+^ B cells), CD3^+^ T cells and CD20^+^ B cells. Kaplan–Meier estimates of OS for peritumoral TIL (both CD3^+^ T cells and CD20^+^ B cells), CD3^+^ T cells and CD20^+^ B cells. No association was observed between immune subpopulations and OS. Kaplan–Meier estimates of OS for PD-L1^+^ and PD-1^+^ cells. No association was observed between cells expressing PD-1 or PD-L1 and OS. The arrow indicates the 3-year survival rate.

The present study scored the density of global tumor-infiltrating lymphocytes (TIL; both CD3^+^ T cells and CD20^+^ B cells), CD3^+^ T cells, and CD20^+^ B cells as a percentage of the intratumoral and peritumoral areas, in both cohorts (Figure 3A). The IHC analysis shows a higher density of TIL in the peritumoral area compared to the intratumoral area in 37 cases, and equally represented between CD3^+^ T cells and CD20^+^ B cells (Table A1). The prevalence of aggregates and TLS was low in the examined tissue fragments of the melanoma metastases. No significant differences were observed between cohort-1 and cohort-2. The density of TIL was significantly correlated with the density of CD3^+^ T cells and CD20^+^ B cells, in both areas (Table A2). However, the density of CD3^+^ T cells and CD20^+^ B cells were not correlated to each other. The Kaplan–Meier curves show that a higher intratumoral and peritumoral TIL, CD3^+^ T-cell, and CD20^+^ B-cell presence, except for intratumoral CD20^+^ B cells, were significantly associated with a longer RFS (Figure 3B–G). There was no association observed between immune subpopulations and OS (Figure A2).

### 3.6. PD-1 and PD-L1 Expression in Melanoma Metastases

**Figure 4:**
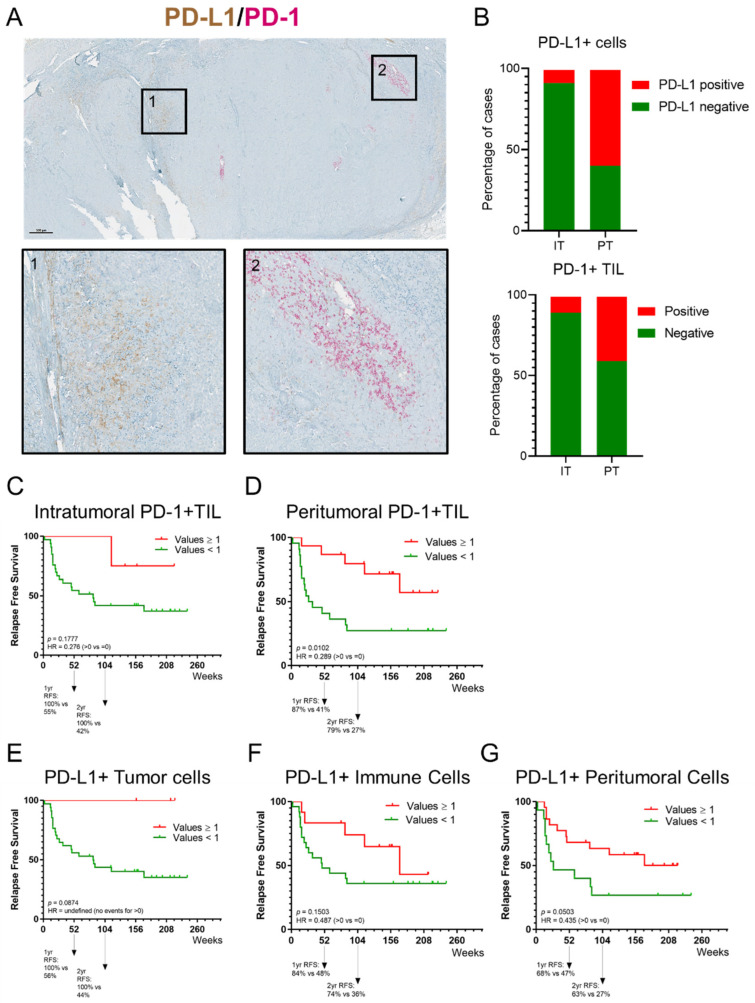
Prevalence of PD-1 and PD-L1 expression on cells in melanoma metastases and their potential correlation with the immune infiltrate evaluated using a dual IHC stain. (**A**) Representative image of a tissue section of a melanoma metastasis stained for PD-1 and PD-L1; shown here are an overview of the section and two regions of interest. Region 1 shows PD-L1^+^ cells; region 2 shows peritumoral PD-1^+^ TIL. IHC slides were scanned at ×40 magnification and the images are displayed at ×2.2 (Scale bar = 500 µm) and ×10 magnification. (**B**) Box plot displaying the percentage of intratumoral and peritumoral PD-1^+^ TIL and PD-L1^+^ cells. A percentage of 59.5% and 40.5% of melanoma metastases, respectively, contained PD-L1^+^ cells and PD-1^+^ TIL in the peritumoral area (threshold of ≥1% positive cells). PD-L1^+^ cells and PD-1^+^ TIL in the intratumoral area were detected in only 8.1% and 10.8%, respectively. (**C**,**D**) Kaplan–Meier estimates of RFS. Presence of peritumoral PD-1^+^ TIL was significantly associated with a longer RFS; intratumoral PD-1^+^ TIL were not associated with improved RFS. (**E**–**G**) Kaplan–Meier estimates of RFS. No significant association in RFS was observed for PD-L1^+^ tumor cells, and PD-L1^+^ peritumoral cells.

The prevalence of PD-1 and PD-L1 expression on cells in melanoma metastases and their potential correlation with the immune infiltrate were evaluated using a dual IHC stain on consecutive sections of FFPE blocks (Figure 4). The analyses revealed that PD-1^+^ TIL and PD-L1^+^ cells (melanoma cells and immune cells, including lymphocytes and myeloid cells) were principally located in the peritumoral area (Figure 4A). Using a threshold of ≥1% positive cells, 59.5% and 40.5% of melanoma metastases contained PD-L1^+^ cells and PD-1^+^ TIL in the peritumoral area, respectively (Figure 4B). PD-L1^+^ cells and PD-1^+^ TIL were detected in only 8.1% and 10.8% of cases in the intratumoral area, respectively. A positive correlation between the density of peritumoral PD-1^+^ TIL and peritumoral CD3^+^ T cells has been observed (Table A2). Moreover, the presence of PD-L1^+^ tumor cells and peritumoral cells were significantly correlated with the density of CD3^+^ T cells (Table A1). The presence of peritumoral PD-1^+^ TIL was significantly associated with a longer RFS; however, intratumoral PD-1^+^ TIL were not associated with improved RFS (Figure 4C,D). A trend towards the presence of PD-L1^+^ tumor cells, and PD-L1^+^ peritumoral cells and longer RFS was observed (Figure 4E–G). There was no association observed between cells expressing PD-1 or PD-L1 and OS (Table A2).

### 3.7. BRAF^V600^/NRAS^Q61/G12/G1 3^ Mutant Circulating Tumor DNA

In total, 36 patients (cohort-1 *n* = 32; cohort-2 *n* = 4) were tested for ctDNA at baseline. Only one patient in cohort-1 had a detectable level of *BRAF^V600^* mutant ctDNA at baseline and relapsed 33 weeks after treatment initiation. Initially, this female, at diagnosis 51-year-old patient presented with two macro-metastatic lymph nodes in the inguinal region. After surgical excision of the primary melanoma situated on the left upper thigh and an inguinal and iliac CLND she started treatment within our clinical trial. The patient relapsed after having received four study drug administrations, eight months after commencement of study treatment. She was diagnosed with diffuse metastases to the lung, liver, lymph nodes, and muscles, indicative of an aggressive disease behavior. She was subsequently treated with standard dosing of pembrolizumab monotherapy to which she did not respond, followed by the combination of dabrafenib and trametinib, and at subsequent progression with ipilimumab monotherapy at standard dosing (one administration) after which the patient died due to progressive disease.

## 4. Discussion

In this single-center, open-label, non-randomized dual cohort phase II clinical trial in patients with resected stage III or IV melanoma an experimental treatment regimen using a fixed low dose of the anti-PD-1 mAb nivolumab administered for one year was associated with encouraging efficacy and safety while a shorter combination regimen, including a single fixed dose of the anti-CTLA-4 mAb ipilimumab, resulted in a higher rate of adverse events and no indication for improved efficacy.

Imbalances between the baseline prognostic characteristics of both cohorts preclude making formal comparisons between them with respect to disease outcome measurements. However, not only did cohort-2 obtain a 12-months RFS-rate that can be considered positive according to the predefined statistical hypothesis (despite the premature recruitment stop to this cohort), but the treatment was also better tolerated. The short combination regimen with a low dose of ipilimumab and nivolumab was found to be inferior in terms of RFS and was also more toxic. Both cohorts had worse baseline prognostic characteristics in comparison to the subjects enrolled in the EORTC 18071 phase III trial [4]. Of note, in contrast to the CheckMate 209–238 and EORTC 18071 phase III trials our trial did not exclude patients with stage III nodal disease who did not have a CLND. In our trial, 59% of the patients in cohort-1 and 41% in cohort-2 had a CLND at baseline. Comparison of survival outcome measures of our small phase II populations with the outcome in reference phase III trials needs to be interpreted with great caution. Nevertheless, RFS from cohort-2 appears very similar to that of nivolumab monotherapy arms in the CheckMate 209–238 and 209–915 phase III trials. We report an estimated 12-months RFS-rate of 85.7% (95% CI, 70–100) while the 12-months RFS-rate was 70.5% (95% CI, 66–74) for the nivolumab treatment-arm in the CheckMate 209–238 trial [3]. The survival of patients treated on cohort-1 is less favorable. Considering the failure of the more toxic ipilimumab plus nivolumab combination regimen in the CheckMate 209–915 phase III trial, there is no place for this combination regimen in the adjuvant treatment of melanoma. However, a randomized, double-blind, placebo-controlled phase II trial suggested that also an adjuvant treatment with nivolumab plus ipilimumab increases RFS significantly in patients with stage IV melanoma with no evidence of disease [33].

Notwithstanding the lower doses used in this trial, both regimens were associated with the occurrence of irAE, expected to occur with an active immunotherapy. The percentage of patients experiencing any grade irAE, which were, respectively, 79% and 86% in cohort-1 and cohort-2, are comparable with the 85–86% incidence reported for nivolumab monotherapy in the CheckMate 209–238 and 209–915 phase III trials [7]. The incidence of grade 3 irAE, which was, respectively, 9% and 5% in cohort-1 and cohort-2, compares favorably with the 13–14% reported in the phase III trials. The same trends are found when compared to adjuvant pembrolizumab treatment in the EORTC 1325 phase III trial [4]. Although the small number of patients treated on our phase II trial prevents us from making a formal comparison, a trend towards a higher incidence of grade 3 irAE is seen with the ipilimumab plus low dose nivolumab regimen. However, the incidence is considerably lower as compared to the incidence of grade 3 irAE in the nivolumab plus ipilimumab arm of the CheckMate 209–915 phase III trial (33%) [7]. This is also in line with the correlation observed between the dose of ipilimumab and the incidence of grade ≥3 toxicity in neo-adjuvant trials [34,35].

Preliminary real-world outcome data of adjuvant treatment with nivolumab in patients with stage III melanoma have shown effectiveness with a 12-months RFS-rate of 83.3% and therefore align with outcomes in the CheckMate 209–238 study [36]. Interestingly, patients treated in this prospective study discontinued treatment more frequently due to irAE; merely 22% of the study population completed study treatment, indicating that a shorter course of treatment could result in comparable effectiveness [36]. Additional real-life studies report premature discontinuation of adjuvant therapy, in most cases due to toxicity, but still resulting in RFS-rates comparable to previous trials [37,38].

Detectable levels of *BRAF^V600^*-mutant ctDNA at baseline in patients with a *BRAF^V600^* mutation but no evidence of disease on medical imaging at baseline (e.g., after CLND or sentinel node procedure) should rise attention to early disease recurrence. However, in our study the low number of patients found to have a detectable ctDNA signal in the blood withholds us from drawing any conclusions on its utility.

In our study, the extent and composition of the immune infiltrate in melanoma metastases was evaluated on dual IHC-stained sections using a previously published methodology [32]. This approach was shown to produce accurate and reproducible scoring by experienced immunopathologists of TIL and TLS in the tumor microenvironment [39]. In our study population, the presence of peritumoral PD-1^+^ TIL was significantly associated with a longer RFS whereas intratumoral PD-1^+^ TIL were not associated with improved RFS. A higher presence of intratumoral and peritumoral TIL, CD3^+^ T cells, and CD20^+^ B cells, except for intratumoral CD20^+^ B cells, was significantly associated with longer RFS. There was no association observed neither between immune subpopulations and OS, nor between PD-1/PD-L1 expression and OS. Biomarker analysis in the CheckMate 238 study revealed that higher levels of IFN-γ gene expression profiling, tumor PD-L1 expression, a higher CD8^+^ T cell infiltrate in tumors and tumor mutational burden (TMB) were associated with favorable RFS and OS with both nivolumab and ipilimumab as an adjuvant treatment [40].

Recently, the application of ICI as a neoadjuvant treatment strategy has gained much interest for the treatment of melanoma with macro-metastases to the locoregional lymph nodes [41]. Although there is particular attention to the combination of nivolumab and ipilimumab in patients with stage III melanoma with macro-metastases, there are currently various other investigated treatment combinations ongoing [42]. Such a neoadjuvant approach offers the possibility to assess the pathological response and to detect possible mechanisms of resistance. The combination of neoadjuvant ipilimumab plus nivolumab induced a 2-year RFS-rate of more than 80%; however, at the cost of a high incidence of irAE [34]. Importantly, the majority of stage III melanoma patients will present with microscopic disease and the neoadjuvant approach will possibly be limited to only a subgroup of patients. Hence, many patients will still rely on adjuvant treatment after surgery in order to reduce their risk for recurrence.

Anti-PD-1 ICI and BRAF-/MEK-inhibition with the dabrafenib plus trametinib (restricted to patients with *BRAF^V600^*-mutant melanoma) have become standard adjuvant treatment options that significantly improve RFS in patients with resectable stage III/IV disease. Ensuring the quality of life of patients by optimizing the currently approved regimens and ensuring affordable access to these treatments becomes an important objective. In particular, younger patients who pursue their professional occupation during adjuvant therapies could benefit from shorter, less toxic, and equally efficacious treatment regimens. Importantly, these patients are at risk of developing long-term sequelae from irAE such as endocrinopathies (e.g., hypothyroidism, adrenal insufficiency) and this risk is correlated with their time of exposure to ICI.

As the indications for ICI are expanding, the financial burden for the health systems worldwide will also increase, and access to treatment with ICI is still limited in many countries. The use of low but possibly effective dosing of available anti-PD-1 ICI in the adjuvant setting could potentially result in a lower financial burden, at the same time allowing more patients to potentially benefit from ICI therapy in countries where access is limited [43]. Recently published data from a phase II trial that investigated a fixed dose of nivolumab (40 mg every 2 weeks) in 30 patients with relapsed or refractory Hodgkin lymphoma resulted in a promising objective response rate, indicative that the utility of low-dose regimens may expand beyond the indication of adjuvant melanoma therapy [44]. Hence, our investigated regimens could be economically advantageous alternatives for patients in countries that do not have access to standard regimens. In addition, low doses of ICI could be of interest for clinical trials investigating combinations with other immunotherapeutic agents that are not sponsored by pharmaceutical companies [45,46].

With respect to the future, new combinations with novel agents, such as the anti-lymphocyte activation gene-3 (LAG-3) mAb relatlimab, and potentially also the neo-adjuvant treatment of patients with nodal macro-metastases will be of importance in shaping the field of adjuvant melanoma treatment. In addition, in such new combinations and regimens, utility of cost-friendly low-dose anti-PD-1 therapy may offer options to patients that have no access to standard doses.

## 5. Conclusions

Our investigated adjuvant regimens with low dose nivolumab have an acceptable safety profile and interesting survival rates in patients with resected stage III or IV melanoma, resembling those of standard regimens. A low dose nivolumab regimen could be an economically advantageous alternative for patients without access to standard regimens.

## Figures and Tables

**Table 1:** Demographic and Clinical Characteristics of the Patients at Baseline.

Characteristic	Cohort-1	Cohort-2
	(*n* = 34)	(*n* = 21)
Sex	Number (%)	
Female	19 (56)	11 (52)
Male	15 (44)	10 (48)
Age	Median (range)	
	54 (29–77)	53 (36–78)
Disease Stage (AJCC 8th edition)	Number (%)	
IIB	1(3)	0 (0)
IIIA	0 (0)	5 (24)
IIIB	21 (62)	5 (24)
IIIC	9 (26)	10 (48)
IV	3 (9)	1 (4)
*BRAFV600* mutation status	Number (%)	
Mutant	15 (44)	5 (24)
Wild type	17 (50)	14 (67)
Unknown	2 (6)	2 (9)
Number of positive lymph nodes	Number (%)	
0	10 (29)	1 (4)
1	11 (32)	12 (57)
2	10 (29)	5 (24)
>2	3 (9)	2 (15)
Nodal metastatic burden	Number (%)	
Microscopic	2 (6)	14 (67)
Macroscopic	17 (50)	5 (24)
In transit/satellite without metastatic nodes	12 (35)	2 (9)
NA	3 (9)	0 (0)
Primary tumor ulceration	Number (%)	
With ulceration	11 (32)	5 (24)
Without ulceration	10 (30)	11 (52)
Unknown	13 (38)	5 (24)

Microscopic: non-clinically detected disease; macroscopic: clinically detected disease Abbreviations: AJCC: American Joint Committee on Cancer; NA: not applicable.

**Table 2:** Adverse Events.

Event	Cohort-1 (*n* = 34)		Cohort-2 (*n* = 21)	
	Any Grade	Grade 3 or 4	Any Grade	Grade 3 or 4
	Number of Patients with Event (%)			
Any adverse event	30 (88)	5 (15)	19 (91)	2 (10)
Treatment-related adverse event	27 (79)	3 (9)	18 (86)	1 (5)
Fatigue	18 (53)	0 (0)	10 (48)	0 (0)
Pruritus	7(21)	0 (0)	8 (38)	0 (0)
Rash	8 (24)	0 (0)	9 (43)	1 (5)
Vitiligo	1 (3)	0 (0)	1 (5)	0 (0)
Pneumonitis	3 (9)	2 (6)	0 (0)	0 (0)
Hyperthryoidism	3 (9)	0 (0)	1 (5)	0 (0)
Hypothyroidism	6 (18)	0 (0)	2 (10)	0 (0)
Hypophysitis *	0 (0)	0 (0)	2 (10)	0 (0)
Sarcoid-like syndrome	0 (0)	0 (0)	1 (5)	0 (0)
Hepatitis	0 (0)	0 (0)	4 (19)	0 (0)
Xerostomia	2 (6)	0 (0)	2 (10)	0 (0)
Dry eyes	0 (0)	0 (0)	1 (5)	0 (0)
Eosinophilia	0 (0)	0 (0)	3 (14)	0 (0)
Arthritis	1 (3)	1 (3)	0 (0)	0 (0)
Any adverse event leading to discontinuation	3 (9)		10 (48)	
Treatment-related adverse event leading to discontinuation	3 (9)		8 (38)	

* clinically presented as adrenal insufficiency.

## Data Availability

The data presented in this study are available on reasonable request from the corresponding author. The data are not publicly available due to ethical/privacy reasons.

## Appendix A

**Table A1:** Immune characteristics of melanoma metastases.

Immune Cell and PD-L1/PD-1 Scoring	Cohort		Total	*p*-Value
	Cohort-1	Cohort-2		
	(*n* = 26)	(*n* = 11)		
% TIL Intratumoral				0.1565 ^1^
*n*	26	11	37	
Mean (SD)	13.6 (15.17)	25.5 (24.82)	17.2 (19.01)	
Median	8.0	20.0	10.0	
Range	0.0, 50.0	1.0, 80.0	0.0, 80.0	
% TIL Peritumoral				0.3949 ^1^
*n*	26	11	37	
Mean (SD)	34.0 (26.84)	44.4 (27.55)	37.1 (27.09)	
Median	22.5	50.0	40.0	
Range	0.0, 80.0	2.0, 80.0	0.0, 80.0	
% CD3^+^ T cells Intratumoral				0.2151 ^1^
*n*	26	11	37	
Mean (SD)	8.4 (9.04)	12.8 (10.93)	9.7 (9.70)	
Median	5.0	10.0	5.0	
Range	0.0, 30.0	0.5, 30.0	0.0, 30.0	
% CD3^+^ T cells Peritumoral				0.8412 ^1^
*n*	26	11	37	
Mean (SD)	24.3 (16.65)	26.9 (21.12)	25.1 (17.83)	
Median	25.0	20.0	25.0	
Range	1.0, 70.0	0.0, 60.0	0.0, 70.0	
% CD20^+^ B cells Intratumoral				0.2756 ^1^
*n*	26	11	37	
Mean (SD)	6.5 (10.89)	16.7 (24.60)	9.5 (16.52)	
Median	2.5	5.0	5.0	
Range	0.0, 50.0	0.0, 80.0	0.0, 80.0	
% CD20^+^ B cells Peritumoral				0.3095 ^1^
*n*	26	11	37	
Mean (SD)	18.8 (20.99)	29.0 (28.20)	21.8 (23.44)	
Median	10.0	15.0	10.0	
Range	0.0, 70.0	0.0, 80.0	0.0, 80.0	
Density of aggregates (Nb/mm^3^)				0.1210 ^1^
*n*	26	11	37	
Mean (SD)	0.4 (0.98)	0.0 (0.15)	0.3 (0.83)	
Median	0.0	0.0	0.0	
Range	0.0, 4.0	0.0, 0.5	0.0, 4.0	
Density of TLS (Nb/mm^3^)				0.4223 ^1^
*n*	26	11	37	
Mean (SD)	0.0 (0.05)	0.0 (0.01)	0.0 (0.04)	
Median	0.0	0.0	0.0	
Range	0.0, 0.2	0.0, 0.0	0.0, 0.2	
% PD-L1^+^ Tumor Cells				0.9162 ^1^
*n*	26	11	37	
Mean (SD)	0.8 (3.92)	0.5 (1.51)	0.7 (3.37)	
Median	0.0	0.0	0.0	
Range	0.0, 20.0	0.0, 5.0	0.0, 20.0	
% PD-L1^+^ Peristromal Cells				0.4786 ^1^
*n*	26	11	37	
Mean (SD)	4.2 (7.75)	4.2 (4.19)	4.2 (6.83)	
Median	1.0	3.0	2.0	
Range	0.0, 35.0	0.0, 10.0	0.0, 35.0	
% PD-L1^+^ Immune Cells				0.1866 ^1^
*n*	26	11	37	
Mean (SD)	0.7 (1.61)	2.1 (3.14)	1.1 (2.23)	
Median	0.0	0.0	0.0	
Range	0.0, 5.0	0.0, 10.0	0.0, 10.0	
% PD-1^+^ TIL Intratumoral				0.3881 ^1^
*n*	26	11	37	
Mean (SD)	0.2 (0.61)	0.3 (0.65)	0.2 (0.62)	
Median	0.0	0.0	0.0	
Range	0.0, 3.0	0.0, 2.0	0.0, 3.0	
% PD-1^+^ TIL Peritumoral				0.4103 ^1^
*n*	26	11	37	
Mean (SD)	1.9 (3.51)	2.5 (4.44)	2.1 (3.76)	
Median	0.0	1.0	0.0	
Range	0.0, 12.0	0.0, 15.0	0.0, 15.0	
FirstRelapse, nb events				
No	9	7	16	
Yes	17	4	21	
SurvStat, nb events				
Alive	19	11	30	
Dead	7	0	7	

^1^ Wilcoxon rank sum *p*-value.

**Table A2:** Immune variables correlations.

Variable		% Intratumoral T Cells	% Peritumoral T Cells	% Intratumoral B Cells	% Peritumoral B Cells	% Intratumoral TIL	% Peritumoral TIL	% PD-L1^+^ TC	% PD-L1^+^ PC	% PD-L1^+^ IC	% Intratumoral PD-1^+^ TIL	% Peritumoral PD-1^+^ TIL
% Intratumoral T cells	Correlation	1.0000										
	*p*-value											
% Peritumoral T cells	Correlation	0.7943	1.0000									
	*p*-value	0.0000										
% Intratumoral B cells	Correlation	0.2683	0.1069	1.0000								
	*p*-value	0.1084	0.5290									
% Peritumoral B cells	Correlation	0.2138	0.2901	0.8032	1.0000							
	*p*-value	0.2039	0.0815	0.0000								
% Intratumoral TIL	Correlation	0.6694	0.4706	0.8768	0.7185	1.0000						
	*p*-value	0.0000	0.0033	0.0000	0.0000							
% Peritumoral TIL	Correlation	0.5965	0.6895	0.6313	0.7961	0.7679	1.0000					
	*p*-value	0.0001	0.0000	0.0000	0.0000	0.0000						
% PD-L1^+^ TC	Correlation	0.4282	0.4038	−0.0099	−0.0079	0.2209	0.2149	1.0000				
	*p*-value	0.0082	0.0132	0.9536	0.9630	0.1889	0.2016					
% PD-L1^+^ PC	Correlation	0.4151	0.3523	0.0170	0.0256	0.1675	0.3873	0.4136	1.0000			
	*p*-value	0.0106	0.0325	0.9204	0.8807	0.3216	0.0179	0.0109				
% PD-L1^+^ IC	Correlation	0.3026	0.3889	−0.1237	−0.0557	0.0435	0.2508	0.3170	0.6181	1.0000		
	*p*-value	0.0687	0.0174	0.4657	0.7433	0.7985	0.1343	0.0559	0.0000			
% Intratumoral PD-1^+^ TIL	Correlation	−0.0271	0.2130	−0.1322	−0.0072	−0.1260	0.1684	−0.0659	−0.0285	0.1226	1.0000	
	*p*-value	0.8736	0.2056	0.4353	0.9660	0.4576	0.3190	0.6985	0.8668	0.4698		
% Peritumoral PD-1^+^ TIL	Correlation	0.0810	0.4186	−0.0093	0.2020	0.0461	0.4208	−0.1180	0.0184	0.3009	0.7554	1.0000
	*p*-value	0.6338	0.0099	0.9566	0.2306	0.7865	0.0095	0.4867	0.9138	0.0703	0.0000	
*p*-value: Ho: corr = 0 H1: corr not equal to 0, two-sided												
Highlighted in yellow, when *p*-value is ≤ 0.05												
*n* = 37 for all correlation												
